# Genome-Wide DNA from Degraded Petrous Bones and the Assessment of Sex and Probable Geographic Origins of Forensic Cases

**DOI:** 10.1038/s41598-019-44638-w

**Published:** 2019-06-03

**Authors:** Daniel Gaudio, Daniel M. Fernandes, Ryan Schmidt, Olivia Cheronet, Debora Mazzarelli, Mirko Mattia, Tadhg O’Keeffe, Robin N. M. Feeney, Cristina Cattaneo, Ron Pinhasi

**Affiliations:** 1 School of Archaeology and Earth Institute, University College of Dublin, Dublin 4 Belfield, Ireland; 2 Department of Evolutionary Anthropology, University of Vienna, Althanstraße 14 1090, Wien, Austria; 3 CIAS, Department of Life Sciences, University of Coimbra, 3000-456 Coimbra, Portugal; 4 LabAnOF, Sezione di Medicina Legale, Dipartimento di Scienze Biomediche per la Salute, Università degli Studi di Milano, Via Mangiagalli 37, 20133 Milano, Italy; 5 School of Medicine, Health Sciences Centre, University College Dublin, Dublin 4 Belfield, Ireland

**Keywords:** Biological techniques, Genetics

## Abstract

The acquisition of biological information and assessment of the most probable geographic origin of unidentified individuals for obtaining positive identification is central in forensic sciences. Identification based on forensic DNA, however, varies greatly in relation to degradation of DNA. Our primary aim is to assess the applicability of a petrous bone sampling method in combination with Next Generation Sequencing to evaluate the quality and quantity of DNA in taphonomically degraded petrous bones from forensic and cemetery cases. A related aim is to analyse the genomic data to obtain the molecular sex of each individual, and their most probable geographic origin. Six of seven subjects were previously identified and used for comparison with the results. To analyse their probable geographic origin, samples were genotyped for the 627.719 SNP positions. Results show that the inner ear cochlear region of the petrous bone provides good percentages of endogenous DNA (14.61–66.89%), even in the case of burnt bodies. All comparisons between forensic records and genetic results agree (sex) and are compatible (geographic origin). The application of the proposed methodology may be a powerful tool for use in forensic scenarios, ranging from missing persons to unidentified migrants who perish when crossing borders.

## Introduction

One of the main focuses of forensic anthropology is to facilitate the personal identification of human remains. Forensic anthropologists assess biological parameters in order to narrow the potential matches and which can also assist in a personal identification (or “positive identification”). The aim of personal identification is to obtain an exact match between postmortem data and the data associated with a specific person, for instance by comparison of specific bony traits with antemortem radiographs (e.g. frontal sinus comparison), surgical implants (e.g. manufacturer information/serial number) and comparison using dental prosthesis or unique dental features. Often, taphonomic alterations heavily damage diagnostic skeletal traits thus limiting the scope and level of confidence of the biological profile which can be generated by the anthropological analysis^1^. Degradation and alteration factors can be physical or biological, including water, temperature, burial depth, acidity, clothing, insect activity, carnivore activity, bodily trauma, as described by Mann *et al*.^2^.

Recently, cutting edge developments in molecular genetics have had a major impact on forensic anthropology and legal medicine; for instance, the identification of human remains on the basis of DNA data obtained from the body which is then compared to various genetic databases of family members of missing persons (e.g. National Missing Persons DNA Database - NMPDD)^3^. Ancient DNA studies which focus on the degradation of DNA are particularly relevant to forensic research since DNA molecules fragments size decrease rapidly after death, with the average length of fragments extracted from hard tissues (bone and teeth) of ancient or sub-ancient human remains ranging between 30–70 base pairs^4–9^, as many factors contribute to the deterioration and fragmentation of postmortem human DNA strands over time, including chemical modifications, microbial activity, temperature, soil acidity, humidity and oxygen levels. In this respect, the DNA analysis of human remains from forensic contexts can benefit from the application of approaches and protocols which are applied routinely in ancient DNA studies.

Methods of ancient DNA sampling, extraction and library preparation protocols, high throughput shotgun sequencing, and the analysis of genome-wide data have dramatically improved during the past decade^8,10^ and to a large extent have replaced previous PCR-based methods. However, while some genetic information can be obtained from historical forensic cases^9^, a major limitation of genome-wide data is that samples with low percentage of endogenous DNA and of low molecular complexity cannot be sequenced to the required depth and coverage in a cost-effective manner, as in many cases >99% of the DNA molecules do not align to the human reference genome and these data must be discarded. Sampling strategy is therefore a key element for obtaining high endogenous DNA yields and the required complexity from degraded and skeletonized human remains^11^. The study by Gamba *et al*.^12^ and Pinhasi *et al*.^13^ have demonstrated that the inner ear cochlear region of the otic capsule of the petrous bone, compared to other skeletal elements, can lead to endogenous ancient DNA yields that are up to 50–100-fold higher than those obtained from other bones, including from remains deposited in regions where environmental conditions are adverse to ancient DNA preservation. In both forensic genetics and ancient DNA research the density of a bone is positively correlated with DNA preservation and sampling is recommended to be carried out whenever possible on dense, weight-bearing bones^14^. The cochlea is enveloped by the densest bone of the otic capsule, and this protection also reduces its postmortem degradation process in comparison to other skeletal elements^15^, and as such is potentially particularly promising for forensic taphonomic investigations (e.g.^15–18^).

Research shows the increasing interest in petrous bones as a strategic region to recover DNA, even though these studies are focused solely on Sequence-Tagged Sites and Short Tandem Repeats (STRs) typing to obtain a match between DNA profiles (DNA profiling). The use of autosomal Single-Nucleotide Polymorphisms (SNPs) in forensic identification is of great relevance especially in the case of forensic DNA phenotyping^19^. A recent study by Kulstein *et al*.^20^ evaluated the potential of the petrous bone for the identification of skeletal remains in eight modern degraded samples. They obtained an adequate amount of genetic material which was sequenced by capillary electrophoresis to identify an STR profile. In addition, the authors investigated phenotyping and biogeographical SNPs (54 biogeographical ancestry SNPs) using Massive Parallel Sequencing (Illumina MiSeq). However, they were not able to compare the actual data on six out of eight samples, as these were unknown subjects. Furthermore, the phenotyping results obtained on the known samples were not always in agreement.

Next Generation Sequencing (NGS), may provide advantages in the use of autosomal SNPs for forensic analysis^19,21^ to perform parallel genotyping of large numbers of SNPs, which can successfully deal with small amounts of degraded DNA^19^, as is shown in the present study.

Concerning forensic degraded samples, a typical taphonomic factor is heat which has shown to degrade DNA molecules. Several studies on the identification of burnt bones have been published^22^. Among these, Fredericks *et al*.^23^, Imaizumi^24^ and Fredericks *et al*.^25^ extracted DNA from burnt bovine compact bones during experimental research, up to a maximum of 250 °C. In these studies, no DNA was recovered from cases with burning temperatures greater than 210 °C. Other research has focused on the analyses of human DNA from samples from forensic casework^6,11,26,27^. Schwark *et al*.^6^ collected several samples from 13 burnt bodies, in attempt to perform STR profiling and mitochondrial DNA (mtDNA) sequencing. Colour grades were used to deduce the approximate burning temperatures. The authors were able to amplify DNA from well preserved and semi-burnt bones using a self-made multiplex PCR system optimized for amplifying highly degraded DNA. However, as noted in Imaizumi^22^, the portion chosen for DNA extraction might have been exposed to a lower temperature than the main area used for classification of burn coloration. Furthermore, in an experiment on a single charred bone (femur), Fondevila *et al*.^27^ suggested that a modified ancient DNA extraction procedure^28^ may allow for an improved DNA typing from degraded skeletal material. In this case-report the authors used mini-STRs and SNPs to confirm identity by DNA profile comparison and represents an interesting application of ancient DNA methods to Forensics. However, for all of these cases studies, the investigators neither sampled petrous bones nor use NGS approaches.

The DNA analysis of burned remains is extremely challenging as the organic components of bone, including DNA molecules, are destroyed at a comparatively early phase in the burning process^22^. Currently, success rates of DNA extraction from burned remains is relatively low, and as such it has a negative impact on the process of identification in forensic casework^22^. The temperature range of the great majority of fire affected forensic cases is 400 °C–1,200 °C ^12,26,29^, as in the three burning cases examined here. The threshold of DNA survival on burnt remains is still not clear and it may significantly change when applying optimal sampling methods, and aDNA/NGS approaches.

The primary aim of our research is to assess, for the first time, the applicability of a specific petrous bone sampling method developed by Pinhasi *et al*.^13^ in combination with a Next Generation Sequencing (NGS) method to evaluate the quality and quantity of DNA obtained from seven forensic cases, including a comparison of burned to non-burned cases. More specifically, we have tested these methods for a number of taphonomic contexts including human remains decomposed in a mountain woodland, modern cemeteries and a tenement, and three cases of burned remains: two were burned in a car and one was burned in a bonfire located in the woods.

All seven cases have an overall poor preservation and are from diverse geographic origins (Italian and international) and varied taphonomic contexts. Six subjects were previously identified (see Material and Methods). These subjects were used as “known” samples to be compare with the results obtained. One subject has not been identified and the analysis conducted here may contribute to the ongoing investigation.

A related aim is the analysis of the obtained genomic data in order to derive 1. the molecular sex of each individual, and 2. their most probable geographic origin based on the analyses on their genetic affinities to genetic data for modern-day populations from various world regions.

## Results

### Taphonomical alterations

In the case of USM 1 and USM 4 (‘USM’ was used as the acronym to identify the collection) the soft tissues were completely charred and the bones were largely charred, with areas of calcination and were heavily affected by thermal destruction (cracking and fragmentation).

In the case of USM 2 the upper part of the subject presented moderate thermal alteration (ranging from no colour changes to charred), while the lower part had severe thermal alteration (ranging from charred to calcined bones). By contrast, USM 3 had no significant taphonomical damage and the degradation of endogenous DNA is related to postmortem decomposition rather than to significant extrinsic environment alteration. USM 6 exhibits severe weathering (cracking, abrasions and bleaching). Similarly, USM 7 and USM 8 display evidence of severe weathering (cracking, abrasion, metal staining and coffin wear^30^). A comprehensive description of the taphonomic alterations of the cranial remains of these subjects is provided in Table 1 (see also Supplementary Fig. S1).

**Table 1:** Details of the taphonomic alteration on the seven cranial samples.

*USM*	Taphonomic details of the cranium
1	*Burnt and fragmented cranium:* charred and fragmented cranial vault. Petrous bones exposed. Anterior cranial base and posterior fossa fragmented. The middle fossa and petrous bones less damaged from heat compared to other areas of the crania. Maceration of remaining soft tissues was not necessary.
2	*Burnt*, *fragmented and incomplete cranium:* cranial vault burnt, fragmented and largely incomplete. Petrous bones exposed. Cranial base complete with intact surface of compact and cortical tissue. Near the squamous portion of the temporal bones the cortical layers were exfoliated with resulting exposure of the diploë. The heat line extended anteriorly to the frontal bone squama, laterally to the base of squamous temporal bones and posteriorly to the cruciform eminence of the occipital bone. In general, the bone tissues are better preserved on the right side of the crania. Maceration of remaining soft tissues was not necessary.
3	*Complete cranium with blunt trauma:* blunt trauma fracture on the right side of the crania with partial exposition of right petrous bone. Non-relevant alteration of bone tissue. Maceration was used to remove the soft tissue.
4	*Burnt and fragmented cranium:* cranial vault extremely fragmented and partially incomplete. Cranial base and petrous bones exposed. Heat line extended superiorly through the orbits and posteriorly towards the occipital squama and laterally to the apex of the mastoid processes. Bone tissue above the heat line is charred with delamination. Maceration was used to remove the soft tissue.
6	*Complete cranium with weathering:* large fracture on the right temporal bone. Right petrous bone partially exposed. Right side and cranial base characterized by sun bleaching and mosaic cracking. Skeletonized remains, maceration of remaining soft tissues was not necessary.
7	*Incomplete cranium with weathering:* cranial base is mostly incomplete. Bone surface is characterized by areas of exfoliation and patches of rough texture. Postmortem open cracks were observed. Skeletonized remains, maceration of remaining soft tissues was not necessary.
8	*Incomplete cranium with weathering:* cranial base and facial bones are fragmented and largely absent. Several postmortem alteration including large and deep exfoliation areas with bone surface generally characterized by fibrous and rough texture. Cancellous bone exposed. Postmortem open cracks were observed. Skeletonized remains, maceration of remaining soft tissues was not necessary.

**Figure 1:**
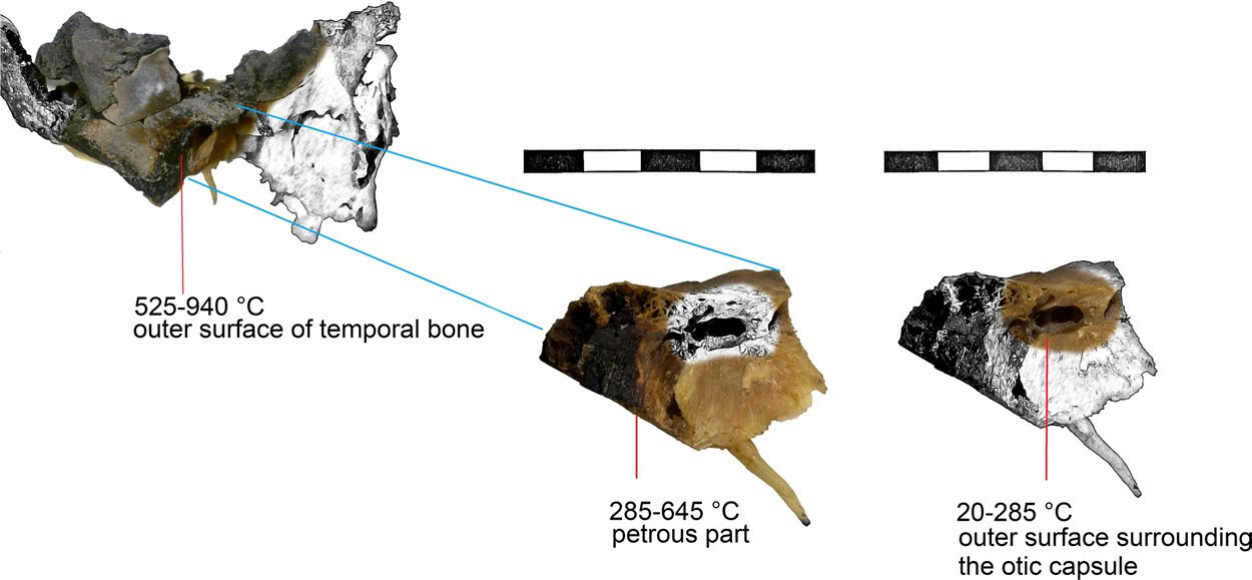
Example of temperature estimation by colour chart: thermal alteration on USM 4 and the temperature estimates in different areas of temporal bone (Shipman *et al*.^31^) (Photo Imaging performed by AKVIS Sketch trial version).

Temperature exposure was estimated based on the assessment of colour changes^31,32^ observed in three samples obtained from individuals affected by thermal alterations (USM 1, USM 2 and USM 4). Table 2 provides colours hues and temperatures estimated in different areas of the same bone (temporal bone) that were used for the estimation of the effective temperature based on Munsell’s colour codes. Exposure of the outer and the inner part of the temporal bones to flames and heat differed, resulting in different coloration in different areas. Coloration was evaluated separately for the outer table of the temporal bone and the inner part (Fig. 1).

**Table 2:** The three thermally altered samples and related Munsell’s codes, colour hues and estimated temperature in different areas of the same bone (see also Figs 1 and 2).

Sample	Munsell’s colours code	Colours	Shipman stage (1984)	Temperature estimation °c (shipman, 1984)	Temperature estimation °c (ellingham, 2015)
**USM 1**					
Temporal bone (outer surface)	7.5YR 8/0, 7/0, 6/0, 5/0, 2.5YR 6/0, 5/0, 2.5Y 7/1, 6/1, N9-5/0,10YR 5/1, 10YR 2/1, 2/2, 10R 1.7/1	Grey, brownish gray, blue grey, black/brownish black and reddish black	3	525–645	500–700
Petrous part of the temporal bone	5Y 8/2, 8/3, 8/4, 7/2, 10YR 4/3, 4/4, 4/6, 10YR 3/1, 3/2, 3/3, 10YR 2/1, 2/2, 10R 2/1	Light gray, pale yellow, yellowish brown, brown, brownish black, dark brown, black, reddish black	2	285–525	200–500
Otic capsule (outer surface)	5Y 8/2, 8/3, 8/4, 7/2, 10YR 4/3, 4/4, 4/6, 10YR 3/1, 3/2, 3/3, 10YR 2/1, 2/2, 10R 2/1	Light gray, pale yellow, yellowish brown, brown brownish black, dark brown, black, reddish black	1	20–285	unburnt to 300
**USM 2**					
Temporal bone (outer surface)	5Y 8/2, 8/3, 84, 7/2, 5Y 8/1, 7/1, 6/1, 5/1, 10YR 5/6, 5/8,10YR 3/1, 3/2, 3/3, 10YR 2/1, 2/2, 10R 2/1	Pale yellow, light gray, gray brown yellowish, brownish black, dark brown, black, reddish black	2/3	285–645	300–600
Petrous part of the temporal bone bone + otic capsule	5Y 8/2, 8/3, 8/4, 7/2, 7/3, 7/4	Light gray, light yellow, pale yellow	1	20–285	unburnt to 300
**USM 4**					
Temporal bone (outer surface)	10YR 4/1, 3/1, 3/2, 3/3, 2.5Y 3/2, 10YR 5/1, 5YR 4/1, 5Y 3/1, 3/2, 2/1, 2/2, 5YR 2/1, 2/2, 10YR 2/1, 2/2	Brownish black, dark, brown, brownish gray, black, olive black	3/4	525–940	400–700
Petrous part of the temporal bone	5Y 8/6, 8/8, 7/6,7/8, 10YR 5/6, 5/8, 10YR 4/3, 4/4, 4/6, 5Y 3/1, 3/2, 2/1, 2/2,10YR 2/1, 2/2, 10R 2/1	Yellow, yellowish brown, brown, olive black, black, reddish black	2/3	285–645	300–500
Otic capsule (outer surface)	5Y 8/6, 8/8, 7/6, 7/8	Yellow	1	20–285	unburnt to 300

**Figure 2:**
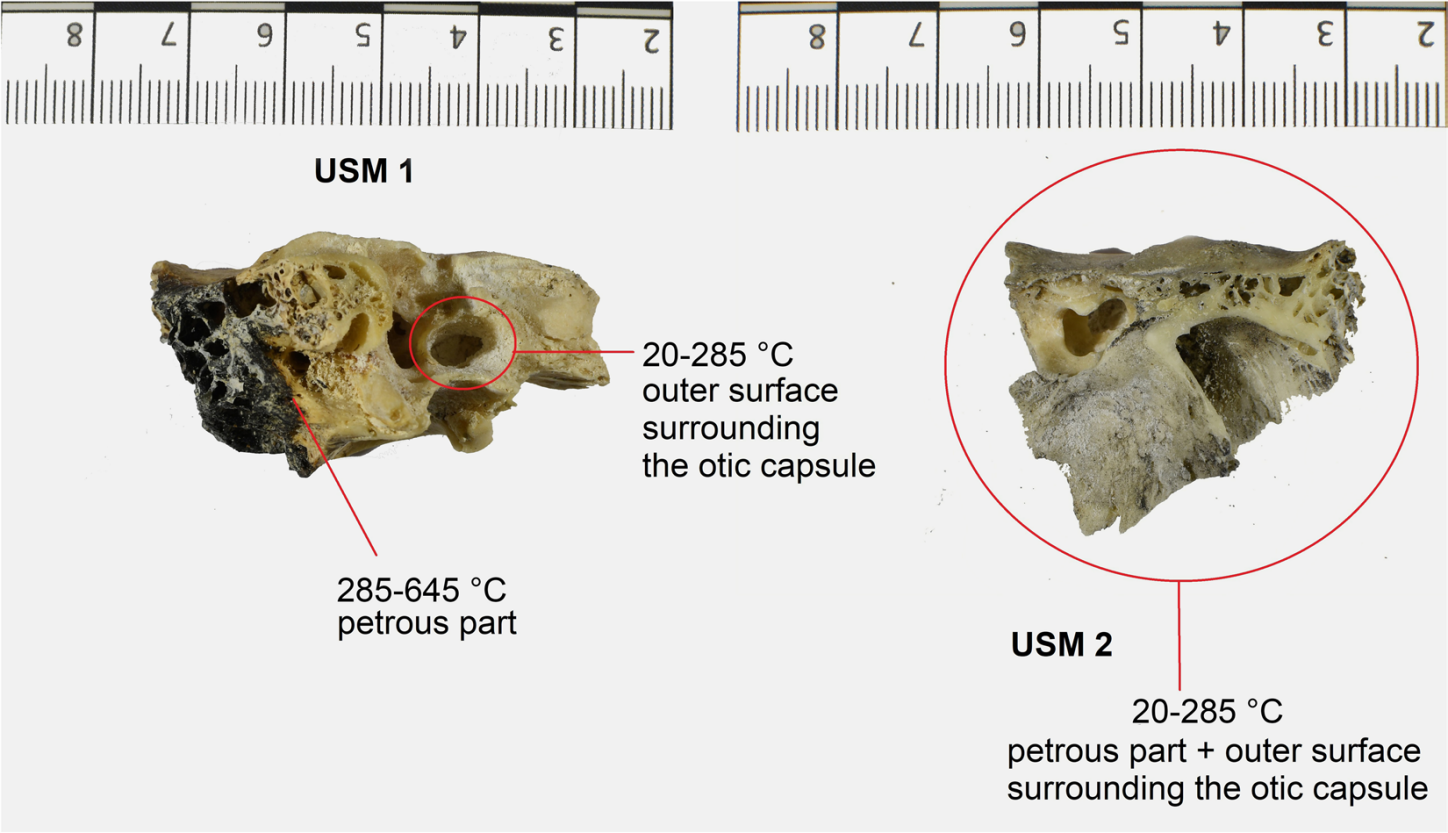
Thermal alteration on USM 1 (left) and USM 2 (right) and the temperature estimates in different areas of temporal bone (Shipman *et al*.^31^). In USM 2 no difference between the petrous part and the specific area around the otic capsule was observed.

The lateral surface of the USM 1 left temporal bone has colours associated with Shipman^31^ stage 3, which corresponds to a stage between 525 and 645 °C, and are attributed to 500–700 °C exposure based on Ellingham *et al*.^29^ In the same sample left petrous part of the temporal bone has colour hues corresponding to Shipman^31^ stage 2 (285–525 °C), implying a 200–500 °C exposure based on Ellingham *et al*.^29^. Finally, the colour of the external lateral surface surrounding the otic capsule of USM 1 corresponds to Shipman^31^ stage 2 (20–285 °C) and between unburnt to 200 °C exposure based on the study by Ellingham *et al*.^29^ (Fig. 2). The coloration of the USM 2 lateral surface corresponds to Shipman^31^ stages 2/3 (285–645 °C) and between 300–600 °C referring to Ellingham *et al*.^29^. The coloration of the internal medial USM 2 left petrous (including the otic capsule area) corresponds to Shipman^31^ stage 1 (20–285 °C) and between unburnt and 300 °C exposure for Ellingham *et al*.^29^ (Fig. 2). In the case of USM 4, the outer part of the right temporal bone colours were assigned to Shipman^31^ stages 3/4 (525–940 °C) implying 400–700 °C exposure referring to Ellingham *et al*.^29^. The USM 4 right petrous has the hues assigned to Shipman^31^ stages 2/3 (285–645 °C) implying 300–500 °C exposure by Ellingham *et al*.^29^. In examining the outer surface around the otic capsule of the same specimen yellow hues (5Y 8/6, 8/8, 7/6, 7/8) were observed, associated with Shipman^30^ stage 1 (20–285 °C) and implying a condition between unburnt to 300 °C exposure based on Ellingham *et al*.^29^.

### DNA analysis

DNA from the petrous bones of these cases was isolated, extracted and prepared as libraries for sequencing on an Illumina platform in a modern molecular DNA lab following established protocols for ancient DNA, with extra precautions to avoid contamination (see “Materials and Methods” section for details). The seven libraries were initially sequenced on the Illumina MiSeq platform. The endogenous human DNA contents ranged between 15–68% and read lengths from 54 ± 10 to 58 ± 10 bp (Table 3). Deamination patterns due to DNA damage varied between 6 and 18% on the 5′ side of the reads, and 5 and 13% on the 3′ side (Table 3), which are consistent with the patterns of damage expected for degraded bones. The negative blanks for the extraction, library, and PCR steps were also sequenced, and yielded very small numbers of human reads (between 63 to 336 reads). All these tests suggested that the DNA retrieved from these individuals was authentic and not contaminated, and so the libraries of these 7 individuals were further sequenced in order to increase coverages.

**Table 3:** Data obtained for each subject after screening on an Illumina MiSeq (Chr. = Chromosomal).

Sample	Total reads	Human reads (hg19)	% endogenous	Average read lengths (stdev)	Deamination frequencies (5′|3′)	Mitochondrial_haplogroup	Chr. Sex
USM-1	317105	108355	34.17	57 (10)	0.06|0.05	U5/B4	M
USM-2	360335	140273	38.93	54 (10)	0.16|0.12	U5a	M
USM-3	355258	243511	68.54	58 (10)	0.08|0.04	H3/H5/H7/H45/H	M
USM-4	376038	139715	37.15	58 (10)	0.09 | 0.06	T2a	M
USM-6	500657	278649	55.66	54 (11)	0.15|0.11	U4b	M
USM-7	451559	273764	60.63	55 (10)	0.17|0.13	U4c/a	M
USM-8	653439	98553	15.08	54 (10)	0.18|0.12	K1a	M

An Illumina NextSeq500 was used to sequence the libraries with single ended read lengths of 75 bp (Table 4). Moderate-to-high endogenous DNA yields (14.61–66.89%) were obtained for all seven petrous bones, including three burnt bones. For these three samples, we obtained 32.33% for USM 1, 36.11% for USM 2 and 36.53% for USM 4. The highest endogenous yield (66.89%) was obtained for USM 3 (the subject recovered decomposed in a tenement), while the lowest yield (14.61%) was obtained for USM 8 (one of the subjects exhumed from modern cemeteries). The average sequence read length from these specific samples was 60 ± 14 bp, which is shorter and with a larger standard deviation than expected in the case of modern samples (e.g. Fernandes *et al*.^9^). Terminal DNA deamination ranged from 7–19% on the 5′ side (C > T) and 5–15% on the 3′ side (G > A) (Table 4). Average contamination estimates that were based on the assessment of mismatches at polymorphic sites on the X chromosome of male individuals^33^ were below 2.78% (0.09% median), and are within the ranges seen in other ancient DNA studies (e.g.^34,35^).

**Table 4:** Data obtained for each subject following Next Generation sequencing (Chr. = Chromosomal).

Sample	Total reads	Human reads (hg19)	% endogenous	Average read lengths (stdev)	Deamination frequencies (5′|3′)	X-chromosome contamination (angsd-c, mom)
USM-1	41043246	13267614	32.33	62 (13)	0.07|0.05	0.0193–0.0228
USM-2	39147452	14137599	36.11	57 (14)	0.17|0.15	0.0000–0.0096
USM-3	41642903	27854992	66.90	63 (14)	0.09|0.06	0.0000–0.0032
USM-4	39373772	14383249	36.53	63 (14)	0.10|0.07	0.0036–0.0088
USM-6	48073336	25789059	53.65	58 (14)	0.16|0.13	0.0000–0.0088
USM-7	48333549	27977434	57.88	58 (14)	0.17|0.15	0.0071–0.0122
USM-8	36557401	5343117	14.62	57 (14)	0.19|0.15	0.0094–0.0278
**Sample**	**X-chromosome contamination** (**angsd-r**)	**Autosomal coverage**	**Snps hits on human origins**	**Mitochondrial coverage**	**Mitochondrial haplogroup**	**Chr**. **Sex**
USM-1	0.0128–0.0182	0.2674X	168656	26.8424X	H-152/H3/H69	M
USM-2	0.0105–0.0105	0.2617X	159523	29.1164X	U5a1	M
USM-3	0.0040–0.0073	0.5753X	304171	45.7935X	H3c2	M
USM-4	0.0061–0.0123	0.2967X	178990	27.0566X	T2a1a	M
USM-6	0.0106–0.0131	0.4883X	269557	29.0281X	U4b1a/b	M
USM-7	0.0078–0.0088	0.5289X	285902	33.7184X	U4c1/U4a2	M
USM-8	0.0000–0.0141	0.0997X	68434	8.6922X	K1a/b/c	M

**Figure 3:**
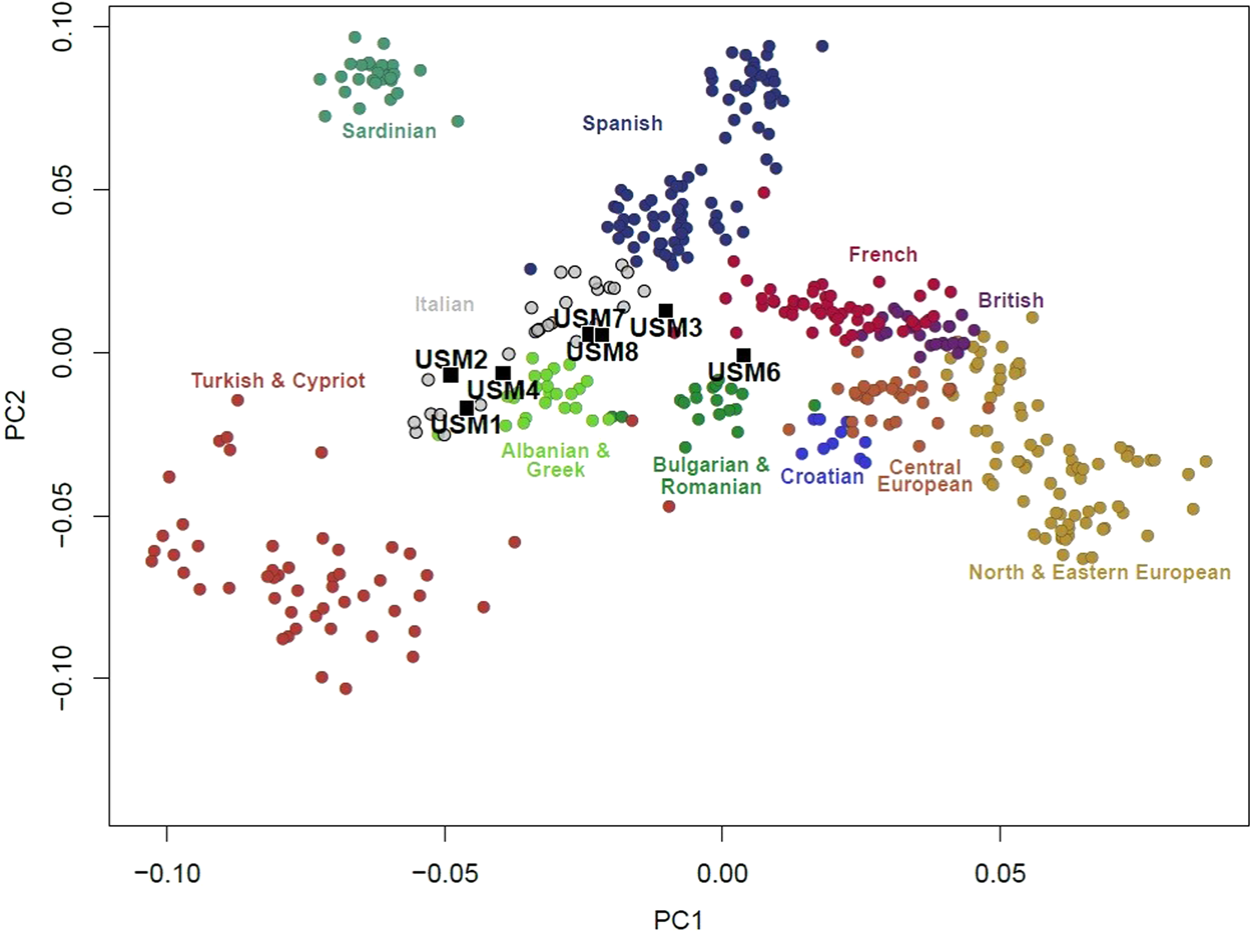
PCA plots of the samples to specific populations.

Sex was correctly attributed for each sample (Tables 3–5). In order to analyse the probable geographic area of origin of each individual, samples were genotyped for the 627.719 SNP positions of the Human Origins dataset from Lazaridis *et al*.^36^ or bases with mapping quality above 30. When coverage for a site was above 1x we randomly selected one of the alleles. Due to the low-coverage of the data we then duplicated the chosen allele to produce pseudo-haploid calls. Then, using the “smartpca” tool from the EIGENSOFT software we projected our individuals on a Principal Component Analysis (PCA) onto the variation of modern humans from the Human Origins dataset. We were able to associate them to specific populations or broad genetic geographical regions, with at least three groupings being visually identified. USM 1, USM 2 and USM 4 cluster close to each other within one end of the Italian cluster that corresponds to individuals from the southern areas of the country, including the island of Sicily. USM 3, USM 7 and USM 8 are positioned within and next to the Italian cluster, but more on a central location occupied by central and northern Italians. Specifically, USM 3 seems to be positioned slightly outside the Italian cluster being pulled in the direction of the French cluster, which could indicate a different genetic affinity but still within South and Western Europe variation. Individual USM 6 clusters away from all other individual and between Balkan and French populations (Fig. 3). We also used mtDNA haplogroup determination to identify the broad geographic area, as several mtDNA haplogroups show restricted continental distributions^37^, with the results showing haplogroups commonly found in Europe. This analysis enabled the correct identification of the most probable geographic origin for the majority of the individuals, which was in agreement with the available forensic data (as shown in detail in Table 5).

**Table 5:** A comparison between sex and the probable geographic region of origin, inferred from the genetic data and the forensic data.

Sample	Sex and geographic origin from SNPs analysis	Sex and geographic origin from forensic data
USM-1	Male, Italian	Male, Italian
USM-2	Male, Italian	Male, Caucasoid (no other information available)
USM-3	Male, South/Southwestern Europe	Male, Portuguese
USM-4	Male, Italian	Male, Italian
USM-6	Male, Central Europe	Male, Swiss
USM-7	Male, Italian	Male, Italian
USM-8	Male, Italian	Male, Italian

## Discussion and Conclusions

The use of human bone sampled from forensic cases and from a variety of taphonomic conditions results in a limited availability of samples. However, the here-presented results demonstrate the viability of obtaining substantial levels of endogenous DNA for forensic cases when targeting the cochlear region of the petrous bone, for cases in which there is extensive degradation to hard tissues as well as heat damage. For the studied cases it has been possible to obtain the sex and probable geographic affinity for these cases with low coverage shotgun genomic data.

We advocate the sampling strategy as a fundamental variable in the genetic analysis of degraded skeletal remains. Furthermore, we support the cochlea in the dense region of the petrous bone as the “target” skeletal element. Our experiment confirms that the application of this process is strategic for forensic studies in regard to three aspects:

1. A moderate to high percentage of endogenous DNA, which has also been reported based on experiments with DNA sampling of petrous bones (Pinhasi *et al*.^13^).
2. The particular location of the otic capsule inside the petrous pyramid, which is in turn located within the cranial cavity, leads to overall better preservation of the DNA. Moreover, in the case of heat damage, it is exposed to lower temperature ranges than more outer parts.
3. The otic capsule is one of the densest parts of the human skeleton contained in the similarly robust petrous bone. These protected and dense bony inner ear structures therefore allow for better preservation^38,39^ and reduced exposure to contamination^40^ compared to more superficial and less dense elements.

The importance for sampling of burned remains is discussed below.

In general, the results show that the highest percentage of endogenous DNA yields (66.89%) were obtained for USM 3, where environmental factors did not affect the subject, as the body was recovered in a highly decomposed state inside an apartment. The lowest yield (14.61%) was obtained from USM 8, the fully skeletonized remains in which the bones, including the cranium, were heavily degraded. This low percentage can be explained by the particular conditions of the cranium: the cranial base is largely absent and deep exfoliation areas were noticed. This degree of deterioration of bone tissue may be linked to the extreme fragmentation of DNA. However, a good percentage of endogenous DNA yields was obtained from USM 6 (53.64%) and USM 7 (57.88%), both of which represent degraded skeletal remains. These contrasting findings are in line with the results obtained in a study of mitochondrial DNA data from postcranial bones^11^ (performed on skeletal remains from the Voegtly Cemetery, in use between 1833 and 1861), that showed no significant correlation between skeletal weathering and DNA quantity, probably because the visual appearance of the bone itself is not a useful predictor of DNA typing results^11^.

The results from the three cases of burned remains are encouraging. Although this sample size is small, it is suggestive of a scenario in which burnt petrous bones and the application of Next Generation sequencing can provide valuable genomic data in case of burned human remains. In a forensic setting, there are several scenarios in which DNA analysis from burned bones is necessary: building fires, explosions, transport incidents, concealed bodies (in an attempt to prevent identification and recovery), among other examples. DNA analysis on burned bones is often challenging and the need for a DNA profile for identification is so great that, as reported by Latham^41^, any available biological element will be processed in the hope of obtaining good quality genetic information. However, as mentioned in the Introduction, the temperature threshold(s) of DNA survival in burned bones is not yet clearly established. Schwark *et al*.^6^ successfully amplified DNA from modern bones burned at temperature that reached over 500 °C, while other studies^22,25,26^ reported severe DNA degradation after short periods of thermal exposure with lower temperature. For instance, Imaizumi *et al*.^24^ reported that in their experiment (using non-human bones) the DNA could not be amplified after 45 minutes exposure at 200 °C. A similar temperature was identified as limit for DNA amplification by Fredericks *et al*.^25^.

One of the most challenging scenarios to anthropologists is the analysis of cremated remains (no soft tissues survive and hard tissues are extremely altered and fragmented). In the forensic field, this kind of investigation is related to the concealment of human remains in homicide cases^42^, or disputes over commercial cremations^43^. In archaeological contexts the analysis of cremations are common, as this practice is an integral part of many funerary rituals^44^. In light of the temperature thresholds mentioned above, the amplification of DNA from cremated remains is extremely difficult, if not impossible. Temperature ranges depend on cremation conditions^45^, in contemporary commercial cremations the temperature may vary between 500 °C and 1,000 °C^43^, for instance. Experimental research indicates that above 500 °C collagen is completely degraded, and beyond 800 °C calcination occurs^25^. A recent study^46^ performed on seven ancient cremated human petrous bones (Iron Age samples) failed to identify human DNA on all seven samples. Of additional importance, a major limitation to this variety of research is that it is difficult to replicate real conditions with actualistic burning experiments^29^.

Despite all these limitations, our findings suggest that the otic capsule, and in particular the cochlea, is the preferable region of the human skeleton to obtain ancient DNA genomic data from burned remains. On the basis of all factors available (exterior surface of temporal bones, characteristics of the post cranial bones and conditions of the crime scenes), USM 4 is the subject that was exposed to the highest temperature (525–940 °C) while USM 2 was most likely exposed to the lowest (285–645 °C). Using the sampling strategy described here, in all three cases we obtained endogenous DNA that, in terms of authenticity and quality, is comparable to the non-burned individuals (Table 4).

Based on the temperatures estimated by assessing colour changes and focusing solely on the otic capsule, it is evident that the samples were exposed to lower heat: around 285–525 °C relative to the petrous bone and 20–285 °C with regard to the otic capsule in USM 1; 20–285 °C for both petrous and otic capsule in USM 2, and finally 285–645 °C for the petrous bone and 20–285 °C for the otic capsule of USM 4. These results suggest that the inner cranial regions may better preserve and protect genetic material from the effects of direct flames and high heat than outer areas; possibly in relation to the fact that they are protected by the brain, neck, cervical spine and the facial skeleton^47^.

Of additional relevance, the genome-wide SNP data obtained here by Next Generation Sequencing enabled the correct identification of the most probable geographic origin for the known individuals tested, in agreement with the forensic data available (detailed in Table 5). As a supplementary test, we examined a femoral sample, and despite the high estimated contamination (Supplementary Tables S1 and S2) the genetically estimated biogeographical origin exactly matched the known origin (See Supplementary Fig. S2 and Supplementary Table S3).

Specifically, the individuals of Italian origin were best detected. For the remaining subjects, the geographic assessment based on the genomic data is more general but in agreement with the known attributions. A more detailed attribution may be possible using more comprehensive datasets that include higher number of individuals and a wider geographical coverage. As mentioned earlier, one subject (USM 2) remains unidentified and unclaimed. In this case we know, on the basis of the genomic analysis, that USM 2 is a Caucasian male, but we do not have information on his exact region of origin. Our results indicate the origin of USM 2 is in the southern areas of Italy, including the island of Sicily. In this specific case, the results obtained here can contribute useful information to forensic investigations.

Taphonomic processes can severely alter the human remains and the traditional morphometric traits used for the estimation of an individual’s biological profile (including sex, age, stature and ancestry). While our results are only based on seven forensic cases, they suggest that relevant information can be obtained from this approach, including sex and the geographic origin of unknown human remains.

The application of the methodology proposed here to narrow down the pool of potential candidates in the search for identity could be a powerful tool for use in forensic scenarios, ranging from missing persons to unidentified migrants who die when crossing borders (e.g. between Mexico and the United States and between Africa and the Middle East on the way to Europe). In the latter case, in 2014 for instance, as many as 3,000 migrants died or disappeared while trying to migrate to Europe^48^. A consideration to bear in mind, however, is that genome-wide SNP data does not represent information on political nationality, and therefore should be carefully contextualized, case by case, to avoid serious misunderstandings during investigation.

A further note of caution relates to the sample tested here. As previously mentioned, on account of the fact that the subjects were selected from real forensic cases in order to obtain as realistic conditions as possible, the relatively few numbers of cases to which our methodology was applied and the lack of controls on the study variables examined warrant that results be treated as preliminary. The potential of this approach, which appears very promising, should be further explored on a larger sample.

Ultimately, the protected and dense inner ear portion of the petrous bone can provide a good amount of endogenous DNA in forensic samples, including those exposed to extreme taphonomic conditions, as in the case of burned human bodies. The combined approach by the application of the Next Generation sequencing can therefore provide an opportunity to create a basis for personal identification in challenging forensic contexts.

## Materials and Methods

We examined seven contemporary individuals selected from the skeletal remains collection housed in the University of Milan. Six subjects are “unclaimed identified”, in which the identity of the individual is known yet relatives did not claim the body. USM 1 was previously identified by comparing dental features between those of the remains and a presumed decedent (in accordance with the biological profiles). USM 3 was previously identified by comparing biological profiles and permanent tattoos observed on soft tissues both on the victim and the “potential match”; in this specific case, tattoos were used as physical evidence. USM 4 was identified by comparing specific dental device and USM 6 by genetic analysis using conventional DNA profiling of the remains and the presumed decedent.

USM 7 and USM 8 represent two subjects that were separately exhumed from modern cemeteries, selected from the University of Milan CAL (Collezione Antropologica Labanof^49,50^) collection, which included subjects who died during 1990 and were exhumed from the Cemetery of Milan. All antemortem data (including death certificates) are available for these subjects.

**Figure 4:**
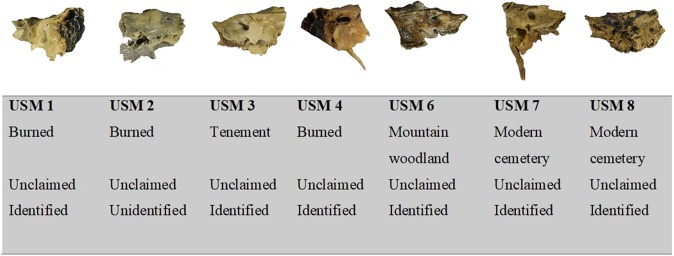
The seven petrous bone recovered in different taphonomic contexts used in this study.

One subject (USM 2) remains unidentified and unclaimed (see Fig. 4). The information available were gathered using standardized bioanthropological analysis of the skeletal elements.

The seven subjects originated from different geographical areas, and were recovered from different taphonomic contexts (see Fig. 4). USM 1 and USM 4 were found as two separate cases burnt in separate cars. USM 2 was found in a bonfire located in a woodland. USM 3 was recovered decomposed in a tenement. USM 6 was found in a mountainous woodland environment. USM 7 and USM 8 were exhumed, as previously mentioned, and selected for analysis on account of the particularly degraded nature of the remains.

The estimation of the temperature exposure of the thermally altered bones was assessed by colour changes, following^31^ and based on the Munsell Colour Chart^32^, and also revised by Ellingham *et al*.^29^.

### Sampling for DNA analysis

We sampled the petrous portions of the left or right temporal bone of each individual. As a preliminary cleaning step, the petrous bone samples were immersed in 5% bleach for 30 seconds, rinsed with ethanol and then dried for approximately one hour. Following this, we obtained 50 mg of fine bone powder from the cochlea by means of direct drilling (Dremel 9100 Fortiflex rotary tool, fitted with a small-sized spherical 1.5 mm grinding bit) from the inferior side of the petrous portion^51^ and decontaminated with bleach and ethanol. This procedure is minimally destructive; visible damage is circa 3 mm in diameter. Following this, the bone powder was collected in a 1.5 mL sterile Eppendorf tube. This procedure was carried out in a sample preparation facility at the Ardmore Laboratory, University College Dublin, Ireland.

### Extraction, DNA library preparation and Next-Generation sequencing

In order to reduce potential contamination of the samples, all following steps were performed while using face masks, and all reagents and consumables were thoroughly sterilized via exposure to short-wave ultraviolet light, bleach, or both, before leaving the clean room facilities.

DNA was extracted using the Dabney protocol^8^ with a Roche High Pure Extender Tube. Approximately 50 mg of bone powder was combined with 1 mL of an extraction buffer solution containing 0.5 M EDTA and Proteinase K (Roche Diagnostics). After vortexing, the bone powder was incubated at 37 °C with rotation for 18 hours in a ThermoMixer C. Thereafter 13 mL of binding buffer was added to the Roche Assembly Tubes. The 1.5 mL tubes with powder were centrifuged for 2 min at 13,000 rpm, the supernatant transferred to the 13 mL of binding buffer, and then the spin columns were centrifuged. After a dry spin for 1 min at 6,000 rpm, 650 μL of PE washing buffer was added to the spin column and centrifuged again for 1 min at 6,000 rpm. The flow-through was discharged and the washing step repeated. Afterwards, the spin column was dried (spun for 1 min, at maximum speed), placed into a clean Eppendorf 1.5 mL tube and eluted by adding 25 µL of TET buffer to the silica membrane and incubated for 10 minutes at 37 °C and centrifuged at maximum speed for 30 s. This step was repeated to obtain a total of 50 µL DNA extract.

We carried out library preparation steps following the protocol by Meyer and Kircher^52^, that are specific for Next-Generation sequencing. A Blunt-End Repair (New England Biolabs) master-mix was prepared and mixed with the DNA extract, then incubated for 15 min at 25 °C and 5 min at 12 °C. Double-stranded adapters were ligated to the DNA using T4 DNA Ligase (ThermoFisher Scientific), and incubating the samples for 30 min at 25 °C. These adapter sequences were then filled-in with Bst Polymerase (New England Biolabs), during an incubation step of 30 min at 37 °C, followed by thermal inactivation of the enzyme at 80 °C for 20 min. Accuprime Pfx Supermix (Life Technology) was used to perform indexing PCRs with the universal primer IS4, and a unique indexing primer per sample. 3 μL of the library was added to a freshly prepared PCR mix, resulting in a total volume of 25 μL. PCR amplification was performed using the following temperature cycling profile: 5 min at 95 °C, 12 cycles of 15 sec at 95 °C, 30 sec at 60 °C and 30 sec at 68 °C, followed by a final period of 5 min at 68 °C.

In order to purify PCR reactions MinElute (Qiagen) columns were used. Assessment of the PCR reactions and concentrations of each sample were performed on the Agilent 2100 Bioanalyzer following the guidelines of the manufacturer. Based on the concentrations indicated by the Bioanalyzer, samples were pooled in equimolar ratios. The concentration of the pool was assessed both on a Bioanalyzer and a Qubit, after which it was screened on an Illumina MiSeq platform (65 bp) at the UCD Centre for Food Safety, School of Public Health. Deeper sequencing was further performed on an Illumina NextSeq500 (75 bp) at UCD Conway Institute of Biomolecular and Biomedical Research.

### Bioinformatics analyses

A custom ancient DNA bioinformatics pipeline written by the Pinhasi Lab was applied for processing short length raw MiSeq data. The software cutadapt v1.524 was used to trim adapter sequences^53^. Minimum overlap was set to 1 (- O 1) and minimum length to 17 bp (-m 17). Alignment to the human reference genome (hg19, GRCh37) was processed by the Burrows-Wheeler Aligner v.0.7.5a-r40525 298 with disabled seed (-l 1000) and filtering for reads with a minimum phred quality score of 30^54^. Duplicated sequences were removed using Samtools v0.1.19-96b5f2294a26^54^ and deamination frequencies assessed using the mapDamage tool^55^. X-chromosome contamination was estimated following the approaches applied by^33,56^ and using software ANGSD^57^.

Single nucleotide polymorphisms were called using the Genome Analyzer Tool Kit’s (GATK) v.3.3-0-g37228af Pileup tool for the 354,212 positions present in the Harvard “Fully public genotype dataset” described in 10304^36^.

### Ethics statement

The Italian “Regolamento di Polizia Mortuaria” DPR 285/90 article 40–43 specifies that unclaimed human skeletal remains may be use by hospitals and universities for teaching and academic research, and thus this study was exempt from full ethical review (HS-E-17-65).

## Supplementary information

Supplementary information

## Data Availability

The datasets generated and analysed in the current study are not publicly available due to the medico-legal origin of the samples, but may be made available from the corresponding authors upon reasonable request.
