# Supplementary material for: Genome-Wide DNA from Degraded Petrous Bones and the Assessment of Sex and Probable Geographic Origins of Forensic Cases
Source: Sci Rep. 2019 Jun 3;9:8226. doi: 10.1038/s41598-019-44638-w (PMC6547751; doi:10.1038/s41598-019-44638-w)

Genome-wide DNA from degraded petrous bones and the assessment of sex and probable geographic origins of forensic cases

Daniel Gaudio, Daniel Fernandes, Ryan Schmidt, Olivia Cheronet, Debora Mazzarelli, Mirko Mattia, Tadhg O'Keefe, Robin N. M. Feeney, Cristina Cattaneo, Ron Pinhasi

Supplementary Information

Supplementary Table S1: USM 5, data after screening on an Illumina MiSeq

SAMPLE	TOTAL READS	TRIMMED READS	UNIQUE HUMAN_READS	% ENDOGENOUS	%GC	READ LENGTHS	MAP DAMAGE	MITOCHONDRIAL_ HAPLOGROUP	CHR. SEX
USM-5 MISEQ	105225	70515	38045	36.16	40	58+-10	0.05 | 0.03	J2b	F

Supplementary Table S2: USM 5, data following Illumina NextSeq sequencing

SAMPLE	TOTAL READS	HUMAN READS (HG19)	% ENDOGENOUS	AVERAGE READ LENGTHS (STDEV)	DEAMINATION FREQUENCIES (5' | 3')	X-CHROMOSOME CONTAMINATION (ANGSD-C, MOM)
USM-5 NEXTSEQ	7101617	2400631	33,8040	64 (14)	0.05 | 0.04	0.24

X-CHROMOSOME CONTAMINATION (ANGSD-R)	AUTOSOMAL COVERAGE	SNPS HITS ON HUMAN ORIGINS	MITOCHONDRIAL COVERAGE	MITOCHONDRIAL HAPLOGROUP	CHR. SEX
0.10-0.16	0.0499X	35198	26.2295X	J2b1	F

Supplementary Table S3: A comparison between sex and the probable geographic region of origin, inferred from the genetic data and the actual forensic data in USM 5

Sample	Sex and geographic origin from SNPs analysis	Sex and geographic origin from forensic data
USM-5	Female, Bulgarian/Romanian	Female, Romanian

Supplementary Figure S1: Cranial remains with taphonomic alterations used for this research.

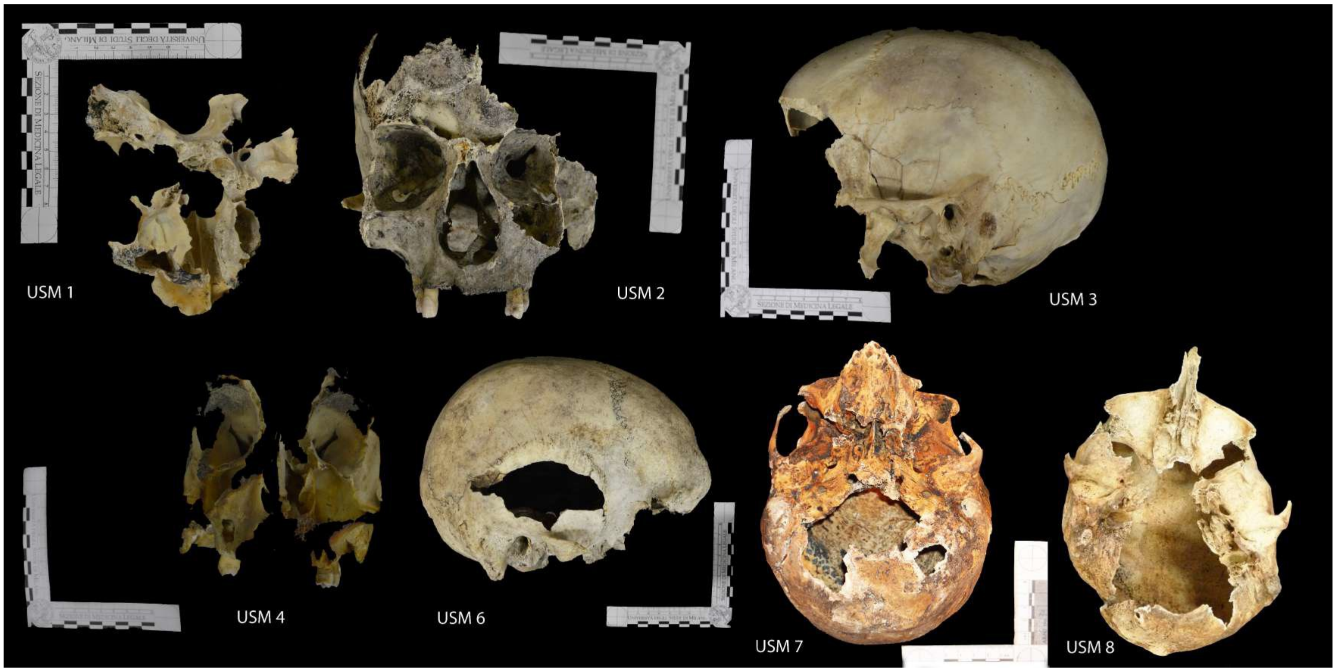

Supplementary Figure S2: PCA plots of the samples, with USM 5 included, to specific populations. USM 5 has a match with Bulgarian and Romanian populations.

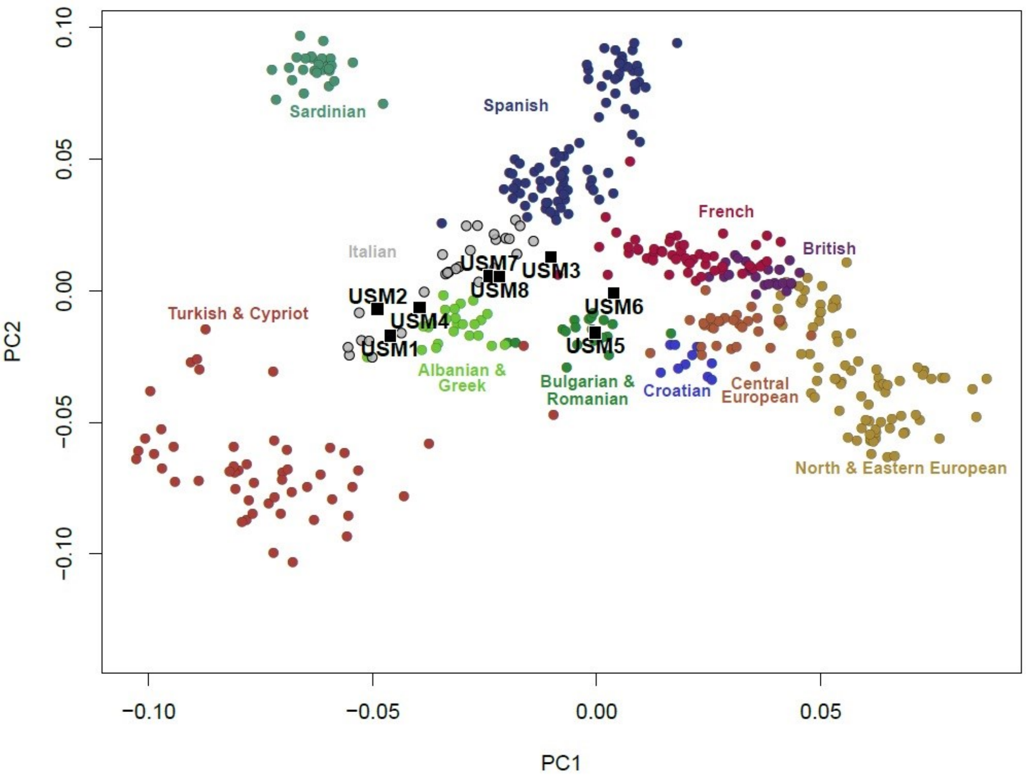

Supplement: Supplementary file 1 — Supplementary information [file 41598_2019_44638_MOESM1_ESM.pdf]
